# Nine New Triterpene Glycosides, Magnumosides A_1_–A_4_, B_1_, B_2_, C_1_, C_2_ and C_4_, from the Vietnamese Sea Cucumber *Neothyonidium* (=*Massinium*) *magnum*: Structures and Activities against Tumor Cells Independently and in Synergy with Radioactive Irradiation

**DOI:** 10.3390/md15080256

**Published:** 2017-08-16

**Authors:** Alexandra S. Silchenko, Anatoly I. Kalinovsky, Sergey A. Avilov, Vladimir I. Kalinin, Pelageya V. Andrijaschenko, Pavel S. Dmitrenok, Ekaterina A. Chingizova, Svetlana P. Ermakova, Olesya S. Malyarenko, Tatyana N. Dautova

**Affiliations:** 1 G.B. Elyakov Pacific Institute of Bioorganic Chemistry, Far Eastern Branch of the Russian Academy of Sciences, Pr. 100-letya Vladivostoka 159, Vladivostok 690022, Russia; sialexandra@mail.ru (A.S.S.); kaaniv@piboc.dvo.ru (A.I.K.); avilov-1957@mail.ru (S.A.A.); pandriyashchenko@mail.ru (P.V.A.); paveldmt@piboc.dvo.ru (P.S.D.); martyyas@mail.ru (E.A.C.); svetlana_ermakova@hotmail.com (S.P.E.); vishchuk87@gmail.com (O.S.M.); 2 A.V. Zhirmunsky Institute of Marine Biology Far East Branch of Russian Academy of Sciences, Palchevsky St. 17, Vladivostok 690041, Russia; tndaut@mail.ru

**Keywords:** *Neothyonidium magnum*, triterpene glycosides, magnumosides, sea cucumber, cytotoxic activity, radioactive irradiation

## Abstract

Nine new sulfated triterpene glycosides, magnumosides A_1_ (**1**), A_2_ (**2**), A_3_ (**3**), A_4_ (**4**), B_1_ (**5**), B_2_ (**6**), C_1_ (**7**), C_2_ (**8**) and C_4_ (**9**) as well as a known colochiroside B_2_ (**10**) have been isolated from the tropical Indo-West Pacific sea cucumber *Neothynidium* (=*Massinium*) *magnum* (Phyllophoridae, Dendrochirotida) collected in the Vietnamese shallow waters. The structures of new glycosides were elucidated by 2D NMR spectroscopy and mass-spectrometry. All the isolated new glycosides were characterized by the non-holostane type lanostane aglycones having 18(16)-lactone and 7(8)-double bond and differed from each other by the side chains and carbohydrate moieties structures. Magnumoside A_1_ (**1**) has unprecedented 20(24)-epoxy-group in the aglycone side chain. Magnumosides of the group A (**1**–**4**) contained disaccharide monosulfated carbohydrate moieties, of the group B (**5**, **6**)—tetrasaccharide monosulfated carbohydrate moieties and, finally, of the group C (**7**–**9**)—tetrasaccharide disulfated carbohydrate moieties. The cytotoxic activities of the compounds **1**–**9** against mouse spleen lymphocytes, the ascites form of mouse Ehrlich carcinoma cells, human colorectal carcinoma DLD-1 cells as well as their hemolytic effects have been studied. Interestingly, the erythrocytes were more sensitive to the glycosides action than spleenocytes and cancer cells tested. The compounds **3** and **7** significantly inhibited the colony formation and decreased the size of colonies of DLD-1 cancer cells at non-cytotoxic concentrations. Moreover, the synergism of effects of radioactive irradiation and compounds **3** and **7**–**9** at subtoxic doses on proliferation of DLD-1 cells was demonstrated.

## 1. Introduction

The triterpene glycosides from sea cucumbers (class Holothurioidea) have a long history of investigation. These marine natural products are characterized by significant structural diversity [1,2,3] and taxonomic specificity which enables their use in resolving some systematic ambiguities [4,5,6]. Additionally, the triterpene glycosides exhibit a wide spectrum of biological activities [7,8], including anticancer effects against different cancer cell lines [9,10,11,12,13,14].

The glycosides from the sea cucumber *Neothyonidium* (=*Massinium*) *magnum* (Phyllophoridae, Dendrochirotida) have been previously investigated. The first studied sample of *N. magnum* was collected near the shores of New Caledonia [15]. The main component of glycosidic fraction, monosulfated tetraoside, “neothyоnidioside”, had the holostane-type aglycone with 9(11)- and 25(26)-double bonds and a 16-keto-group. Another sample of *N. magnum* was collected near Vietnam’s shore [16]. The main component of its glycosidic fraction, neothyonidioside C, was different from “neothyonidioside” and characterized by the C-16-acetylated holostane-type aglycone having 7(8)- and 25(26)-double bonds. The carbohydrate chain of neothyonidioside C had the same set of monosaccharide residues as “neothyonidioside” but was disulfated.

Herein we report the results of investigation of *N. magnum* also collected in Vietnamese shallow waters but having the glycosides, magnumosides A_1_–A_4_ (**1**–**4**), B_1_ (**5**), B_2_ (**6**), C_1_ (**7**), C_2_ (**8**) and C_4_ (**9**), which is significantly different from the compounds isolated previously. The structures of the glycosides were established based on ^1^H and ^13^C NMR spectra and 2D NMR (^1^H,^1^H-COSY, HMBC, HSQC, ROESY) and confirmed by HR-ESI mass spectrometry. The cytotoxic activities of **1**–**9** against mouse spleen lymphocytes, the ascites form of mouse Ehrlich carcinoma cells, mouse erythrocytes, and human colorectal adenocarcinoma DLD-1 cells were tested. The effects of compounds **1**–**9** on proliferation, colony formation of DLD-1 cells as well as the synergism of radioactive irradiation and compounds effects have been studied.

## 2. Results and Discussion

### 2.1. Structural Elucidation of the Glycosides

**Figure 1:**
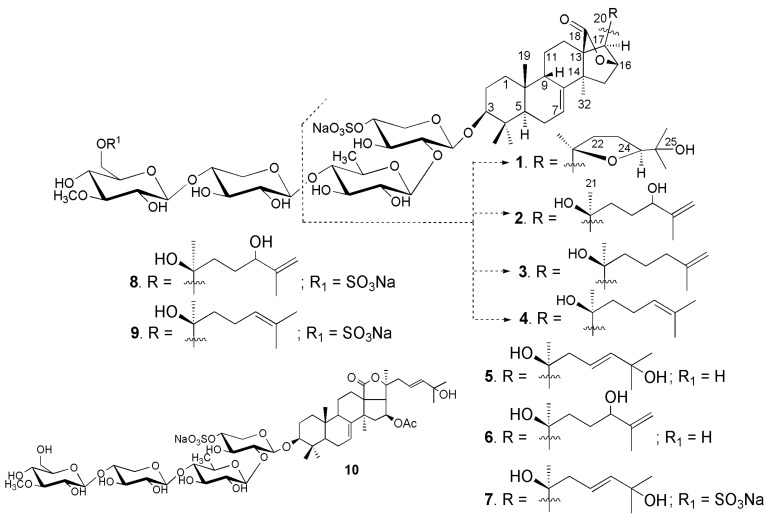
Chemical structure of the glycosides **1**–**10** isolated from *Neothyonidium magnum.*

The sea cucumber *Neothyonidium* (=*Massinium*) *magnum* contains a very complicated mixture of glycosides, thus the isolation of individual compounds was rather labor-consuming and multistage. The concentrated ethanolic extract of *N. magnum* was chromatographed on a *Polychrom-1* column (powdered Teflon, Biolar, Olaine, Latvia). The glycosides were eluted with 50% EtOH and separated by chromatography on Si gel column using CHCl_3_/EtOH/H_2_O (100:100:17) and (100:125:25) as mobile phases. The obtained fractions were subsequently subjected to HPLC on a silica-based Supelcosil LC-Si (4.6 × 150 mm) column, on a reversed-phase semipreparative Supelco Ascentis RP-Amide (10 × 250 mm) column or analytical Diasfer *C-8* (4.6 × 250 mm) column to yield the magnumosides A_1_ (**1**) (3.6 mg), A_2_ (**2**) (5.0 mg), A_3_ (**3**) (3.7 mg), A_4_ (**4**) (8.0 mg), B_1_ (**5**) (2.6 mg), B_2_ (**6**) (1.8 mg), C_1_ (**7**) (5.7 mg) and C_2_ (**8**) (2.5 mg), C_4_ (**9**) (15 mg) and colochiroside B_2_ (**10**) (2.0 mg) (Figure 1). The known compound **10** was identified by comparison of its ^1^H and ^13^C NMR spectra with those reported for colochiroside B_2_ (**10**, 3β-*O*-[3-*O*-methyl-β-d-glucopyranosyl-(1→3)-β-d-xylopyranosyl-(1→4)-β-d-quinovopyranosyl-(1→2)-4-*O*-sodium sulfate-β-d-xylopyranosyl]-16β-acetoxyholosta-25-hydroxy-7,23*E*-diene) from *Colochirus robustus* [17].

The ^1^H and ^13^C NMR spectra of carbohydrate parts of magnumosides A_1_–A_4_ (**1**–**4**) were coincident to each other, indicating the identity of carbohydrate chains of these glycosides. The presence of two characteristic doublets at δ(H) 4.66 (*J* = 7.0 Hz) and 5.00 (*J* = 7.6 Hz) in the ^1^H NMR spectra of the carbohydrate chains of **1**–**4** correlated by the HSQC spectra with the signals of anomeric carbons at δ(C) 104.8 and 105.2, correspondingly, were indicative of a disaccharide chain and β-configuration of glycosidic bonds. The ^1^H,^1^H-COSY and 1D TOCSY spectra of **1**–**4** showed the signals of two isolated spin systems assigned to the xylose and quinovose residues. The positions of interglycosidic linkages were confirmed by the ROESY and HMBC spectra of **1**–**4** (SM, Table 1) where the cross-peaks between H(1) of the xylose and H(3) (C(3)) of an aglycone and H(1) of the quinovose and H(2) (C(2)) of the xylose were observed. Thus, the carbohydrate chains of magnumosides of the group A (**1**–**4**) were identical to those of holothurins of the group B, that are characteristic glycosides for the representatives of the genus *Holothuria* and *Actinopyga* (Holothuriidae, Aspidochirotida) [1,18,19,20].

**Figure 2:**
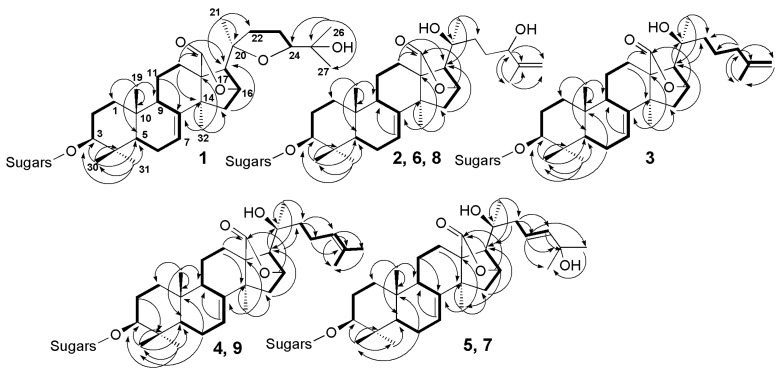
^1^H,^1^H-COSY (**—**) and key HMBC (H→C) correlations for the aglycones of compounds **1**–**9**.

**Figure 3:**
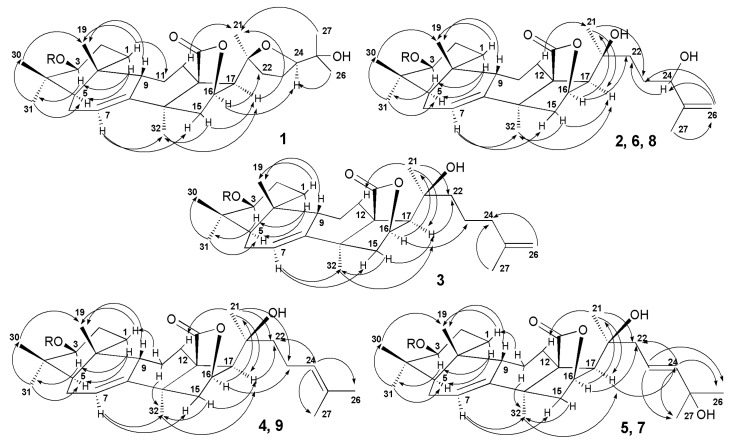
Key ROESY correlations for the aglycones of compounds **1**–**9**.

The molecular formula of magnumoside A_1_ (**1**) was determined to be C_41_H_63_O_16_SNa from the [M_Na_ − Na]^−^ ion peak at *m*/*z* 843.3838 (calc. 843.3842) in the (−)HR-ESI-MS. Analysis of the ^1^H and ^13^C NMR spectra (Table 2 and Table 3) of the aglycone part of magnumoside A_1_ (**1**) suggested the presence of an 18(16)-lactone that was deduced from the characteristic signals of carbons C(18) (δ(C) 180.9) and C(20) (δ(C) 81.9) and characteristic signals of oxygen-bearing methine CH-O(16) (δ(C) 79.6; δ(H) 4.84 (s)). The availability of an 18(16)-lactone and (*S*)-configuration of C(16) asymmetric center was confirmed by the absence of coupling constant *J*_17/16_ for H(17) signal (δ(H) 2.48 (s)) in the ^1^H NMR spectrum of **1** as well as by the presence of correlations H(16)/C(18) in the HMBC spectrum (Figure 2) and H(16)/H(21) in the ROESY spectrum (Figure 3). The signals of olefinic methine group H-C(7) (δ(C) 122.4; δ(H) 5.61 (dt, *J* = 2.3, 7.4 Hz)) and quaternary carbon C(8) (δ(C) 147.6) in the ^13^C- and ^1^H NMR spectra were indicative of 7(8)-double bond in the aglycone nucleus. The signal of oxygenated tertiary asymmetric carbon C(20) assigned by the characteristic for the aglycones of sea cucumber glycosides HMBC correlation H(21)/C(20) was observed at δ(C) 81.9. Its δ(C) value was similar to those values in onekotanogenin [21] and in colochiroside E [22] having the 18(16)-lactone and acetylated C(20) position. However, the presence of acetoxy-group in the aglycone of **1** was excluded by ESI mass spectrometry.

Additionally, there was another downshifted resonance in the ^13^C NMR spectrum of **1** at δ(C) 86.7 corresponding to the oxygen bearing methine carbon located at α-position to hydroxylated C-25. Its position as C(24) was deduced from the ^1^H,^1^H-COSY spectrum where the signals of isolated spin system from H(22) to H(24) were observed (Figure 2). Based on the NMR and ESI-MS data the presence of 20(24)-epoxy-group was suggested. The correlations from H-C(24) (δ(H) 3.97 (dd, *J* = 5.6; 9.3) to C(22), C(23), Me(26), Me(27); from H-C(22) to C(20), Me(21) and from Me(26)(27) to C(24) in the HMBC spectrum were in good agreement with this suggestion. Another downshifted signal (δ(C) 70.1) was assigned to the oxygen-bearing tertiary carbon positioned as C(25) that was deduced from the HMBC correlations Me(26)/C(25) and Me(27)/C(25). The multiplicity of the proton H(24), neighboring it, which was observed as doublet of doublets, confirmed this. The presence in the ^1^H–^1^H-COSY spectrum of the signals of four isolated spin systems confirmed the sequences of protons from H(1) to H(3); from H(5) to H(12); from H(15) to H(17) and finally from H(22) to H(24). The HMBC correlations Me(30)/C(4), Me(31)/C(4), Me(19)/C(10), Me(32)/C(13), C(14) and H(17)/C(20) allowed to elucidate the positions of quaternary carbons in the aglycone polycyclic nucleus.

The ROESY correlations (Figure 3) of **1** H(5)/H(3), H(5)/Me(31); H(9)/Me(19); H(17)/Me(21); (17)/Me(32) and Me(21)/H(12) confirmed the common elucidated earlier for the other sea cucumber glycosides (3*S*,5*R*,9*S*,10*R*,13*S*,14*S*,16*S*,17*R*,20*S*)-configurations [22,23,24] in magnumoside A_1_ (**1**). The ROE correlations H(16)/H(24); H(17)/H(24) and Me(21)/Me(27) observed in the ROESY spectrum of **1** could be realized in the case of (24*S*)-configuration only. Moreover, the coupling constants of H(24) (dd, *J* = 5.6; 9.3 Hz) in the ^1^H NMR spectrum were very close to the calculated ones, based on the dihedral angles values in MM2 optimized model of (20*S*),(24*S*)-isomer of **1**.

The carbohydrate chain structure of **1** was confirmed by the (−)ESI-MS/MS of the [M_Na_ − Na]^−^ ion at *m*/*z* 843.4, where the peaks of fragment ions were observed at *m*/*z* 741.3 [M_Na_ − Na − SO_3_Na + H]^−^ and 697.3 [M_Na_ − Na − Qui + H]^−^. The (+)ESI-MS/MS of the [M_Na_ + Na]^+^ ion at *m*/*z* 889.4 demonstrated the peaks of ions at *m*/*z*: 769.4 [M_Na_ + Na − NaHSO_4_]^+^, 623.4 [M_Na_ + Na − NaSO_4_ − Qui]^+^, 491.3 [M_Na_ + Na − NaSO_4_ − Qui − Xyl]^+^.

**Figure 4:**
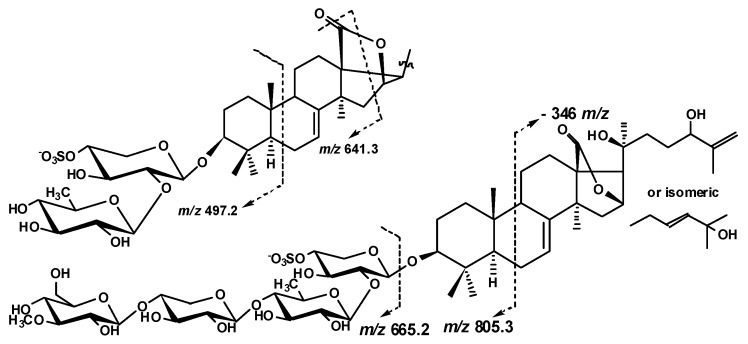
The aglycones fragmentation observed in the (−)ESI-MS/MS of compounds **1**–**9.**

Additionally, an unusual fragmentation pattern, characteristic for **1**–**9**, caused by the presence of 18(16)-lactone ring in their aglycones, was observed. In the (−)ESI-MS/MS of all biosides (magnumosides belonging to the group A) the ion peaks at *m*/*z* 497.2 appeared as a result of cleavage of B-ring of the aglycone, and 641.3 appeared as a result of D-ring cleavage, were observed (Figure 4).

Based on these results, the structure of magnumoside A_1_ (**1**) was determined as 3β-*O*-[β-d-quinovopyranosyl-(1→2)-4-*O*-sodium sulfate-β-d-xylopyranosyl]-9β*H*,20(*S*),24(*S*)-epoxy-25-hydroxylanosta-7-ene-18(16)-lactone.

The molecular formula of magnumoside A_2_ (**2**) was determined to be C_41_H_63_O_16_SNa from the [M_Na_ − Na]^−^ molecular ion peak at *m*/*z* 843.3838 (calc. 843.3842) in the (−)HR-ESI-MS. Extensive analysis of the ^1^H-, ^13^C- (Table 2 and Table 3) and 2D NMR spectra of the aglycone part of magnumoside A_2_ (**2**) showed similarity of its polycyclic system to that of **1**. Indeed, the signals at δ(C) 182.6 (C(18)), 80.1 (C(16)), 62.2 (C(17)) as well as at δ(H) 5.12 (brs, H(16)) and 2.72 (s, H(17)) confirmed the presence of an 18(16)-lactone moiety, the signals at δ(C) 122.3 (C(7)) and 147.6 (C(8)) with corresponding proton signal at δ(H) 5.55–5.57 (m, H(7)) demonstrated the presence of 7(8)-double bond. However the signal of C(20) was high-shifted to δ(C) 71.3 when compared with that of **1** and was close to those of progenins obtained from cladoloside C by its alkaline treatment and having the identical to each other aglycone nuclei with hydroxylated C(20) position and non-shortened side chains [25]. The signals corresponding to the side chain of **2** formed the isolated spin system in the ^1^H,^1^H-COSY spectrum (Figure 2) from H(22) to H(24), indicating the latter signal was down-shifted to δ(H) 4.33 (brt, *J* = 6.0 Hz, H(24)) due to hydroxylation of this position and the multiplicity of the signal showed its vicinity to quaternary carbon C(25). The characteristic signals at δ(C) 148.4 (C(25)) and 110.6 (C(26)) with corresponding signals of olefinic protons at δ(H) 5.16 and 4.87 (each brs, H_2_(26)) indicated the presence of terminal 25(26)-double bond. The HMBC correlations from Me(27) to C(24), C(25) and Me(26) corroborated the structure of the side chain (Figure 2). The ROESY correlations (Figure 3) H-C(5)/H-C(3), Me(31); H-C(9)/Me(19); H-C(16)/Me(21) and H-C(17)/Me(21); H-C(17)/Me(32) confirmed the common (3*S*,5*R*,9*S*,10*R*,13*S*,14*S*,16*S*,17*R*,20*S*)-configurations in lanostane-type aglycone of magnumoside A_2_ (**2**) [22,23,24].

The absolute configuration of C(24) in **2** was determined by modified Mosher’s method. Treatment of compound **2** with (−)-(*R*)- and (+)-(*S*)-MTPA chlorides gave (*S*)- and (*R*)-MTPA esters, correspondingly, and their ^1^H,^1^H-COSY spectra were analyzed. The ∆*^SR^* signs were calculated and their values were negative for H(22) and H(23) and positive for H(26) and H(27), which allowed us to assign the (*24S*) configuration.

The (+)ESI-MS/MS of the [M_Na_ + Na]^+^ ion at *m*/*z* 889.4 demonstrated the peaks of fragment ions at *m*/*z*: 769.4 [M_Na_ + Na − NaHSO_4_]^+^, 623.4 [M_Na_ + Na − NaSO_4_ − Qui]^+^, 491.3 [M_Na_ + Na − NaSO_4_ − Qui − Xyl]^+^, analogous to those for **1**, indicating the identity of their carbohydrate chains. The (−)ESI-MS/MS of **2** demonstrated the peak of fragment ion at *m*/*z* 697.3 [M_Na_ − Na − Qui + H]^−^ as well as the peaks at *m*/*z* 497.2 and 641.3 observed due to the aglycone cleavages mentioned above (Figure 4). Additionally, the peak [M_Na_ − Na − C_6_H_12_O]^−^ was observed at *m*/*z* 743.3 in the (−)ESI-MS/MS of **2** due to the cleavage of the aglycone side chain by C-20–C-22 single bond.

All these data indicate that magnumoside A_2_ (**2**) is 3β-*O*-[β-d-quinovopyranosyl-(1→2)-4-*O-*sodium sulfate-β-d-xylopyranosyl]-9β*H*,20(*S*),24(*S*)-dihydroxylanosta-7,25-diene-18(16)-lactone, having the new aglycone moiety.

Thus, applying Mosher’s method for the aglycone of **2** corroborated the same (24*S*)-configuration in **1** proposed by the ROESY spectrum.

The molecular formula of magnumoside A_3_ (**3**) was determined to be C_41_H_63_O_15_SNa from the [M_Na_ − Na]^−^ molecular ion peak at *m*/*z* 827.3892 (calc. 827.3893) in the (−)HR-ESI-MS. The comparison of ^1^H, ^13^C NMR spectra (Table 2 and Table 3) of its aglycone part with those of magnumoside A_2_ (**2**) showed the closeness of the signals of the polycyclic systems indicating their identity. The signal of C(20) was observed at δ(C) 71.5 indicating the hydroxylation of this position. The protons of the aglycone side chain formed an isolated spin system H(22)/H(23)/H(24)/H(26)/H(27) in the ^1^H,^1^H-COSY spectrum of **3** (Figure 2) that allowed us to elucidate its structure as having a terminal double bond, for which signals were observed at δ(C) 145.9 (C(25)) and 110.3 (C(26)) in the ^13^C NMR spectrum and at δ(H) 4.74 and 4.73 (each brs, H_2_(26)) in the ^1^H NMR spectrum. The ROESY correlations (Figure 3) confirmed the stereo structures of the aglycone polycyclic system and C(20)-chiral center of magnumoside A_3_ (**3**) to be the same as in **2**.

The (+)ESI-MS/MS of **3** demonstrated the fragmentation of [M_Na_ + Na]^+^ ion at *m*/*z* 873.4 resulting in the appearance of ion-peaks at *m*/*z*: 771.5 [M_Na_ + Na − NaSO_3_ + H]^+^, 753.5 [M_Na_ + Na − NaHSO_4_]^+^, 727.3 [M_Na_ + Na − Qui + H]^+^, 607.4 [M_Na_ + Na − NaSO_4_ − Qui]^+^, 475.3 [M_Na_ + Na − NaSO_4_ − Qui − Xyl]^+^, analogous to those for **1** and **2**, indicating the identity of their carbohydrate chains. The characteristic peaks of fragment ions at *m*/*z* 497.2 and 641.3 were also observed in the (−)ESI-MS/MS of **3** (Figure 4).

All these data indicate that magnumoside A_3_ (**3**) is 3β-*O*-[β-d-quinovopyranosyl-(1→2)-4-*O-*sodium sulfate-β-d-xylopyranosyl]-9β*H*,20(*S*)-hydroxylanosta-7,25-diene-18(16)-lactone.

The molecular formula of magnumoside A_4_ (**4**) was determined to be the same as for **3** (C_41_H_63_O_15_SNa) from the [M_Na_ − Na]^−^ molecular ion peak at *m*/*z* 827.3892 (calc. 827.3893) in the (−)HR-ESI-MS. The comparison of ^1^H, ^13^C NMR spectra (Table 2 and Table 3) of the glycosides **3** and **4** revealed the coincidence of the major part of the signals, except the signals assigned to C(23)–C(27). Actually, in the spectra of **4** the signals at δ(C) 125.1 (C(24)), 131.0 (C(25)) with corresponding olefinic proton signal at δ(H) 5.27 (t, *J* = 7.1 Hz, H(24)) were observed indicating the presence of 24(25)-double bond, which was confirmed by the HMBC and ROESY correlations (Figure 2 and Figure 3). Thus, magnumosides A_3_ (**3**) and A_4_ (**4**) are the isomers by the double bond position that was corroborated by their ESI-MS spectra, demonstrating the presence of the ions with the same *m*/*z* values.

The fragmentation patterns in the (+) and (−)ESI-MS/MS of **4** were the same as for **3**, corroborating the identity of their carbohydrate chains and the isomerism of the aglycones.

Thus, magnumoside A_4_ (**4**) is 3β-*O*-[β-d-quinovopyranosyl-(1→2)-4-*O-*sodium sulfate-β-d-xylopyranosyl]-9β*H*,20(*S*)-hydroxylanosta-7,24-diene-18(16)-lactone.

The ^1^H and ^13^C NMR spectra of carbohydrate parts of magnumosides B_1_ (**5**) and B_2_ (**6**) were coincident to each other showing the identity of carbohydrate chains of these glycosides. The presence of four characteristic doublets at δ(H) 4.65–5.21 (*J* = 7.3–8.0 Hz) in the ^1^H NMR spectra of the carbohydrate chains of **5**, and **6** correlated by the HSQC spectra with the signals of anomeric carbons at δ(C) 104.4–104.8 were indicative of a tetrasaccharide chain and β-configuration of glycosidic bonds. The ^1^H,^1^H-COSY and 1D TOCSY spectra of **5** and **6** showed the signals of four isolated spin systems assigned to two xylose, one quinovose and 3-*O*-methylglucose residues. The positions of interglycosidic linkages were elucidated by the ROESY and HMBC spectra of **5** and **6** (Table 1) where the cross-peaks between H(1) of the first (xylose) residue and H(3) (C(3)) of an aglycone, H(1) of the second (quinovose) residue and H(2) (C(2)) of the first (xylose) residue, H(1) of the third (xylose) residue and H(4) (C(4)) of the second (quinovose) residue and H(1) of the terminal (3-*O*-methylglucose) residue and H(3) (C(3)) of the third (xylose) residue were observed. The signals of C(4) and C(5) of the first (xylose) residue were observed at δ(C) 76.0 and 63.9, correspondingly, indicating the presence of a sulfate group at C(4) of the sugar unit, analogically to the carbohydrate chains of magnumosides of the group A (**1**–**4**). The linear tetrasaccharide monosulfated carbohydrate chain of magnumosides of the group B (**5**, **6**), having the xylose as third monosaccharide unit, were found earlier in “neothyonidioside” [15] isolated from another collection of *N. magnum*, also coincided with the sugar moiety of colochiroside B_2_ (**9**) [17] found in the investigated sample of *N. magnum* and are widely distributed in the triterpene glycosides of sea cucumbers of the orders Aspidochirotida [2] and Dendrochirotida [2,8,26,27,28].

The molecular formula of magnumoside B_1_ (**5**) was determined to be C_53_H_83_O_25_SNa from the [M_Na_ − Na]^−^ molecular ion peak at *m*/*z* 1151.4954 (calc. 1151.4950) in the (−)HR-ESI-MS. The polycyclic system and hydroxylated C(20)-position of **5** were identical to those of **2**–**4** that was deduced from the comparison of their ^1^H, ^13^C NMR spectra (Table 2 and Table 3). The olefinic carbons signals observed at δ(C) 142.8 (C(23)), 121.9 (C(24)) with corresponding protons signals at δ(H) 5.92 (d, *J* = 15.8 Hz, H(23)) and 6.13 (dt, *J* = 6.9; 8.0; 15.8 Hz, H(24)) and the signal of oxigenated tertiary carbon at δ(C) 69.9 (C(25)) in the ^13^C and ^1^H NMR spectra of **5**, correspondingly, were indicative for 23(24)*E*-en-25-ol fragment in the aglycone side chain. This structure was confirmed by the HMBC correlations from H-C(22) to C(23) and C(24), from H-C(23) to C(22), C(25), Me(26) and Me(27) and from Me(26) and Me(27) to C(23), C(24), C(25) (Figure 2). The ROESY correlations also corroborated the aglycone structure (Figure 3).

The (−)ESI-MS/MS of **5** demonstrated the fragmentation of [M_Na_ − Na]^−^ ion at *m*/*z* 1151.4. The peaks of fragment ions were observed at *m*/*z*: 975.4 [M_Na_ − Na − MeGlc + H]^−^, 843.4 [M_Na_ − Na − MeGlc − Xyl]^−^, 697.3 [M_Na_ − Na − MeGlc − Xyl − Qui + H]^−^ corroborating the sequence of sugars in the carbohydrate chain of **5**. The cleavage of the ring B of the aglycone led to the appearance of fragment ion-peak at *m*/*z* 805.3 [M_Na_ − Na − C_21_H_30_O_4_]^−^. Its further fragmentation led to the consequent loss of monosaccharide units and the ion-peaks were observed at *m*/*z* 629.2 [M_Na_ − Na − C_21_H_30_O_4_ − MeGlc + H]^−^, 497.2 [M_Na_ − Na − C_21_H_30_O_4_ − MeGlc − Xyl]^−^, 351.1 [M_Na_ − Na − C_21_H_30_O_4_ − MeGlc − Xyl − Qui + H]^−^.

All these data indicate that magnumoside B_1_ (**5**) is 3β-*O*-[3-*O*-methyl-β-d-glucopyranosyl-(1→3)-β-d-xylopyranosyl-(1→4)-β-d-quinovopyranosyl-(1→2)-4-*O*-sodium sulfate-β-d-xylopyranosyl]-9β*H*,20(*S*),25-dihydroxylanosta-7,23*E*-diene-18(16)-lactone.

The molecular formula of magnumoside B_2_ (**6**) was determined to be C_53_H_83_O_25_SNa from the [M_Na_ − Na]^−^ molecular ion peak at *m*/*z* 1151.4945 (calc. 1151.4950) in the (−)HR-ESI-MS that was coincident with the formula of magnumoside B_1_ (**5**). The analysis of the ^1^H, ^13^C NMR spectra of the aglycone part of **6** showed its identity to the aglycone part of magnumoside A_2_ (**2**) (Table 2 and Table 3). So, the glycosides **5** and **6** are the isomers by the positions of the double bond and hydroxyl group in the side chain.

The peaks of fragment ions in the (−)ESI-MS/MS of **6** were observed at the same *m*/*z* values as in the spectrum of **5**, corroborating the identity of carbohydrate chain structures of these compounds. The consequent fragmentation of the ion [M_Na_ − Na − C_21_H_30_O_4_]^−^ at *m*/*z* 805.3 in the spectrum of **6** that led to the loss of the aglycone and the ion-peak [M_Na_ − Na − C_30_H_45_O_5_]^−^ was observed at *m*/*z* 665.2.

Thus, magnumoside B_2_ (**6**) is 3β-*O*-[3-*O*-methyl-β-d-glucopyranosyl-(1→3)-β-d-xylopyranosyl-(1→4)-β-d-quinovopyranosyl-(1→2)-4-*O*-sodium sulfate-β-d-xylopyranosyl]-9β*H*,20(*S*),24(*S*)-dihydroxylanosta-7,25-diene-18(16)-lactone.

The ^1^H and ^13^C NMR spectra of carbohydrate parts of magnumosides C_1_ (**7**), C_2_ (**8**) and C_4_ (**9**) were coincident to each other and to those of neothyonidioside C, isolated earlier from this species [16], that indicated the identity of carbohydrate chains of these glycosides. The presence of four characteristic doublets at δ(H) 4.65–5.12 (*J* = 7.0–7.9 Hz) in the ^1^H NMR spectra of the carbohydrate chains of **7**–**9** correlated by the HSQC spectra with the signals of anomeric carbons at δ(C) 104.3–104.8 were indicative of a tetrasaccharide chain and β-configuration of glycosidic bonds. The positions of interglycosidic linkages were elucidated based on the ROESY and HMBC spectra (SM, Table 1), where the cross-peaks analogical to **5**, and **6** were observed. The differences between the ^13^C NMR spectra of magnumosides of the groups C (**7**–**9**) and B (**5**, **6**) were in the signals of C(5) and C(6) of terminal monosaccharide residue that were observed in the ^13^C NMR spectra of **7**–**9** at δ(C) 75.5 and 67.1, correspondingly, due to the shifting effects of a sulfate group attached to C(6) of 3-*O*-methylglucose unit. Actually, magnumosides of the group C (**7**–**8**) are characterized by the linear tetrasaccharide disulfated carbohydrate chain, which have been found earlier in the glycosides of *Mensamaria intercedens* [29], *Pseudocolochirus violaceus* [30] and *Colochirus robustus* [17], representatives of the family Cucumariidae, order Dendrochirotida.

The molecular formula of magnumoside C_1_ (**7**) was determined to be C_53_H_82_O_28_S_2_Na_2_ from the [M_2Na_ − Na]^−^ molecular ion peak at *m*/*z* 1253.4328 (calc. 1253.4337) in the (−)HR-ESI-MS. The aglycones of magnumoside C_1_ (**7**) and B_1_ (**5**) were identical to each other due to the coincidence of their ^13^C NMR spectra (Table 2 and Table 3) and their carbohydrate chains differed only by the quantity of sulfate groups. The (−)ESI-MS/MS of **7** where the peaks of fragment ions were observed at *m*/*z* 1133.4 [M_2Na_ − Na − NaHSO_4_]^−^**,** 957.4 [M_2Na_ − Na − NaHSO_4_ − MeGlc]^−^,825.4 [M_2Na_ − Na − NaHSO_4_ − MeGlc − Xyl]^−^, 679.3 [M_2Na_ − Na − NaHSO_4_ −MeGlc − Xyl − Qui]^−^ corroborated the structure of carbohydrate chain. The cleavage of the ring B of the aglycone as well as the loss of one of the sulfate groups led to the appearance of fragment ion-peak at *m*/*z* 805.3 [M_2Na_ − Na − NaSO_3_ − C_21_H_30_O_4_ + H]^−^. Its further fragmentation resulted in the loss of the aglycone, the ion-peak at *m*/*z* 665.2 [M_2Na_ − Na − NaSO_3_ − C_30_H_45_O_5_]^−^, followed by the loss of sugar units, *m*/*z:* 533.1 [M_2Na_ − Na − NaSO_3_ − C_30_H_45_O_5_ − Xyl]^−^, 387.1 [M_2Na_ − Na − NaSO_3_ − C_30_H_45_O_5_ − Xyl − Qui]^−^, 255 [M_2Na_ − Na − NaSO_3_ − C_30_H_45_O_5_ − Xyl − Qui − Xyl]^−^. The ion-peak at *m*/*z* 255 [C_7_H_12_O_8_SNa − Na − H]^−^ is also corresponded to the sulfated 3-*O*-methyl glucose residue.

Therefore, magnumoside C_1_ (**7**) is 3β-*O*-[6-*O*-sodium sulfate-3-*O*-methyl-β-d-glucopyranosyl-(1→3)-β-d-xylopyranosyl-(1→4)-β-d-quinovopyranosyl-(1→2)-4-*O*-sodium sulfate-β-d-xylopyranosyl]-9β*H*,20(*S*),25-dihydroxylanosta-7,23*E*-diene-18(16)-lactone.

The molecular formula of magnumoside C_2_ (**8**) coincided with that of **7** (C_53_H_82_O_28_S_2_Na_2_) that was deduced from the [M_2Na_ − Na]^−^ molecular ion peak at *m*/*z* 1253.4341 (calc. 1253.4337) in the (−)HR-ESI-MS. The aglycone of magnumoside C_2_ (**8**) was identical to those of the magnumosides A_2_ (**2**) and B_2_ (**6**) that was suggested based on the coincidence of their ^13^C NMR spectra (Table 2 and Table 3). So, magnumoside C_2_ (**8**) is additionally sulfated, by C(6) of 3-*O*-methylglucose residue, analog of magnumoside B_2_ (**6**). The (−)ESI-MS/MS of **8** corroborated its isomerism to the magnumoside C_1_ (**7**) since their fragmentation patterns were the same and the ion-peaks were characterized by the identical *m*/*z* values.

Thus, magnumoside C_2_ (**8**) is 3β-*O*-[6-*O*-sodium sulfate-3-*O*-methyl-β-d-glucopyranosyl-(1→3)-β-d-xylopyranosyl-(1→4)-β-d-quinovopyranosyl-(1→2)-4-*O*-sodium sulfate-β-d-xylopyranosyl]-9β*H*,20(*S*),24(*S*)-dihydroxylanosta-7,25-diene-18(16)-lactone.

The NMR spectra of the aglycone moiety of magnumoside C_4_ (**9**) were coincident to those of magnumoside A_4_ (**4**) (Table 2 and Table 3).

The molecular formula of magnumoside C_4_ (**9**) was determined to be C_53_H_82_O_27_S_2_Na_2_ from the [M_2Na_ − Na]^−^ molecular ion peak at *m*/*z* 1237.4390 (calc. 1237.4388) in the (−)HR ESIMS. The peaks of fragment ions in the (−) ESI MS/MS of **9** were observed at *m*/*z* 1135.4 [M_2Na_ − Na − NaSO_3_ + H]^−^**,** 1117.4 [M_2Na_ − Na − NaHSO_4_]^−^**,** 959.4 [M_2Na_ − Na − NaSO_4_ − MeGlc]^−^, 827.4 [M_2Na_ − Na − NaSO_4_ − MeGlc − Xyl]^−^, 681.3 [M_2Na_ − Na − NaSO_4_ −MeGlc − Xyl − Qui]^−^ corroborated the structure of carbohydrate chain. The loss of the aglycone from the ion at *m*/*z* 1135.4 [M_2Na_ − Na − NaSO_3_ + H]^−^ led to the appearance of the ion-peak at *m*/*z* 665.2 [M_2Na_ − Na − NaSO_3_ − C_30_H_45_O_4_]^−^, its further fragmentation resulted in the consequent loss of sugar units, *m*/*z*: 533.1 [M_2Na_ − Na − NaSO_3_ − C_30_H_45_O_4_ − Xyl]^−^, 387.1 [M_2Na_ − Na − NaSO_3_ − C_30_H_45_O_4_ − Xyl − Qui]^−^, 255 [M_2Na_ − Na − NaSO_3_ − C_30_H_45_O_4_ − Xyl − Qui − Xyl]^−^. The ion-peak at *m*/*z* 255 [C_7_H_12_O_8_SNa − Na − H]^−^ is also corresponded to the sulfated 3-*O*-methyl glucose residue.

All these data indicate that magnumoside C_4_ (**9**) is 3β-*O*-[6-*O*-sodium sulfate-3-*O*-methyl-β-d-glucopyranosyl-(1→3)-β-d-xylopyranosyl-(1→4)-β-d-quinovopyranosyl-(1→2)-4-*O*-sodium sulfate-β-d-xylopyranosyl]-9β*H*,20(*S*)-hydroxylanosta-7,24-diene-18(16)-lactone.

All the new glycosides, magnumosides A_1_ (**1**), A_2_ (**2**), A_3_ (**3**), A_4_ (**4**), B_1_ (**5**), B_2_ (**6**), C_1_ (**7**), C_2_ (**8**) and C_4_ (**9**), isolated from *N. magnum*, are characterized by the presence of non-holostane-type aglycones with 18(16)-lactone moieties and non-shortened side chains. The aglycone of **1** having 20(24)-epoxy group in the side chain was found first in the triterpene glycosides of sea cucumbers. The aglycones, having 18(16)-lactone, are of rare occurrence in the sea cucumber glycosides. Such type aglycones were found in two variants: with shortened side chains, like in some glycosides from *Eupentacta fraudatrix* [31,32,33] and *Pentamera calcigera* [34]; and, that is more rare, with non-shortened side chains, like in psolusoside B from *Psolus fabricii* [21], colochiroside E from *Colochirus robustus* [22] and in magnumosides A_1_ (**1**), A_2_ (**2**), A_3_ (**3**), A_4_ (**4**), B_1_ (**5**), B_2_ (**6**), C_1_ (**7**), C_2_ (**8**) and C_4_ (**9**).

These aglycones are strongly differing from the holostane-type aglycones found in isolated earlier glycosides, “neothyonidioside” and neothyonidioside C [15,16], from New-Caledonian and Vietnamese collections of *N. magnum*, correspondingly. In contrast, the aglycone of colochiroside B_2_ (**10**) found in the investigated sample of *N. magnum* is biogenetically very close to that of neothyonidioside C which also has the holostane-type aglycone with 16-acetoxygroup and differs in the side chain structure. Furthermore, the monosulfated tetrasaccharide carbohydrate chain of **10** was identical to those of “neothyonidioside” and magnumosides of the group B (**5**, **6**). The disulfated tetrasaccharide chain of magnumosides of the group C (**7**–**9**) was identical to that of neothyonidioside C. The finding of compound **10** in recent Vietnamese collection of *N. magnum* structurally close to found earlier glycosides from this species confirms the correctness of biological identification of the all studied samples. The predominance of non-holostane-type glycosides and the fact that neither “neothyonidioside” nor neothyonidioside C have been found in the studying sample of the animal could be explained by the changes in the quantitative and qualitative composition of different components of glycosidic fraction in the samples of one species collected in different places and seasons. The representative example of such changes demonstrated the components of glycosidic fraction of *Psolus fabricii.* Psolusoside A, a holostane glycoside was predominant in *P. fabricii* collected near the shore of the Onekotan Island of Kuril Ridge and non-holostane psolusoside B having a 18(16)-lactone was the minor one [35]. The opposite situation was observed in samples of *P. fabricii* collected in the Kraternaya Bay of Ushishir Islands of the Kuril Archipelago, where psolusoside A was found only in trace amount and psolusoside B was the predominant component [36].

**Figure 5:**
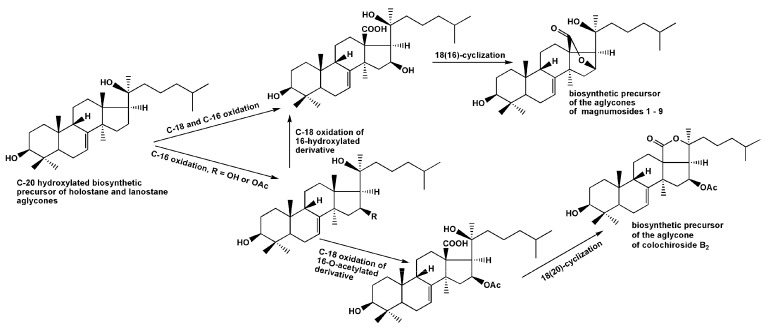
The hypothetic scheme of the aglycones biosynthesis of glycosides of *N. magnum.*

The sets of magnumosides A_2_→B_2_→C_2_ and B_1_→C_1_, in which the glycosides were characterized by the same aglycones and different carbohydrate chains within the set, demonstrated the biosynthetic transformations of the carbohydrate chains resulting in their elongation and additional sulfation. The biosynthetic modifications of the aglycones are mainly concerned with side chains transformations, when the double bonds and hydroxyls are introduced in different positions. The aglycone of magnumosides B_1_ (**5**) and C_1_ (**7**) with 23(24)-double bond and 20,25-hydroxyls is obviously could be the precursor of the unusual aglycone of magnumoside A_1_ (**1**) with 20(24)-epoxy-25-hydroxy-fragment. The similar conversion of the aglycone having a linear side chain with a double bond and hydroxyl group to the aglycone having epoxy-group was observed in the process of obtaining the genins with 18(16)-lactone moieties by chemical transformations of the holostane aglycone of cladoloside C [25]. It was considered as a biomimetic reaction, modeling the biosynthetic process in the glycoside aglycones. The aglycone structure of colochiroside B_2_ (**10**) is biogenetically related with the aglycones of **1**, **5** and **7**. It is known that the formation of 18(16)-lactone biosynthetically occurs in C(18)-carboxylated derivatives having both hydroxylated C(16) and C(20) positions. However, when the further oxidation (acetylation or carboxylation) of C(16) precedes the carboxylation of C(18) the holostane-type aglycones with functionality at C(16) are biosynthesized [37] (Figure 5). In the case of **10,** this process took place. Interestingly, the oxidative transformations of the side chain in biosynthesis of **10** may precede the lactonization.

### 2.2. Biological Activities of Glycosides

#### 2.2.1. Hemolytic and Cytotoxic Activities of the Glycosides **1**–**9** against Mouse Spleenocites and the Ascites Form of Mouse Ehrlich Carcinoma Cells

The cytotoxic action of the compounds **1**–**9** against mouse spleenocytes and the ascites form of mouse Ehrlich carcinoma cells as well as hemolytic action against mouse erythrocytes have been studied (Table 4). Magnumoside A_1_ (**1**) was the only glycoside inactive in all tests that was caused by unusual aglycone structure having 18(16)-lactone in combination with 20(24)-epoxy-25-hydroxy-fragment. Magnumosides A_2_ (**2**), B_1_ (**5**) and B_2_ (**6**) demonstrated moderate hemolytic activity and were non-cytotoxic up to the ultimate investigated concentration of 100 μM, that was explained by the presence of hydroxyls in their side chains [17,32,33]. However, magnumosides C_1_ (**7**) and C_2_ (**8**) that had aglycones identical to those of magnumosides B_1_ (**5**) and B_2_ (**6**) and differed from the latter compounds by the presence of the additional sulfate group were the exclusions from this relation and demonstrated significant effects in all tests. Moreover, magnumosides C_1_ (**7**) and C_4_ (**9**) turned out to be the most active compounds in this series. So, the activity-decreasing influence of hydroxyl-group in the side chain of **7** and **8** was counterbalanced by the additional sulfate group attached to C-6 of terminal monosaccharide residue of these compounds. On the whole, the erythrocytes were more sensitive to the glycosides action than the spleenocytes and cancer cells studied.

#### 2.2.2. The Effect of the Glycosides on Cell Viability of Human Colorectal Adenocarcinoma DLD-1 Cells

To determine cytotoxic effect of the compounds against human colorectal adenocarcinoma, DLD-1 cells were treated with various concentrations of **1**–**9** (0–100 µM) for 24 h and then cell viability was assessed by the MTS assay. It was showed that **1, 2, 4**–**6** did not possessed cytotoxic effect against DLD-1 cells that is in good correlation with the data on Echrlich carcinoma cells. The magnumoside A_3_ (**3**), magnumoside C_1_ (**7**), magnumoside C_2_ (**8**) and magnumoside C_4_ (**9**) decreased cell viability with IC_50_ value of 30.3, 34.3, 32.9, 37.1, and 33.9 µM, respectively (Table 5). Thus, for further experiments we chose the concentrations of investigated compounds lower than IC_50_, at which no significant cytotoxic effect on DLD-1 cells was observed.

#### 2.2.3. The Effect of the Glycosides on Formation and Growth of Colonies of Human Colorectal Adenocarcinoma DLD-1 Cells

**Figure 6:**
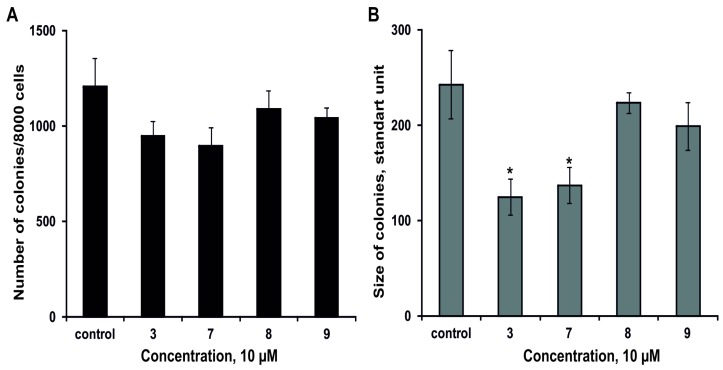
The effect of the glycosides **3**, **7**–**9** on colony formation of DLD-1 cells. (**A**) The compounds decreased the number of colonies of cancer cells. (**B**) The compounds decreased the size of colonies of cancer cells. Data are shown as means ± standard deviation and the asterisks (* *p* < 0.05) indicates a significant decrease in colony formation of cells treated with the compounds compared with the control.

The effect of **3**, **7**–**9** on the colony formation of DLD-1 cells using soft agar assay has been studied. The magnumoside A_3_ (**3**) and magnumoside C_1_ (**7**) inhibited spontaneous colony formation by 22% and 26%, respectively, (Figure 6A) and, at the same time, they reduced colonies size of DLD-1 cancer cells by 49% and 43%, respectively, (Figure 6B). On the other hand, magnumosides C_2_ (**8**) and C_4_ (**9**) possessed slight inhibitory activity in this experiment (the percentages of inhibition were less than 20%) (Figure 6A,B).

#### 2.2.4. The Synergism of Radioactive Irradiation and the Compounds Effects on Proliferation and Colony Formation of Human Colorectal Adenocarcinoma Cells

At first, the individual effect of radiation or the compounds at concentration 2 µM on colony formation of DLD-1 cells was checked. The tested compounds did not influence the process of colonies formation at the dose of 2 µM. The number and size of colonies of DLD-1 cells were found to be decreased by 7% and 67%, respectively, after radiation exposure at dose of 4 Gy. Moreover, the synergism of effects of radioactive irradiation (4 Gy) and the compounds **3, 7**–**9** (2 µM) was not observed (data not shown).

**Figure 7:**
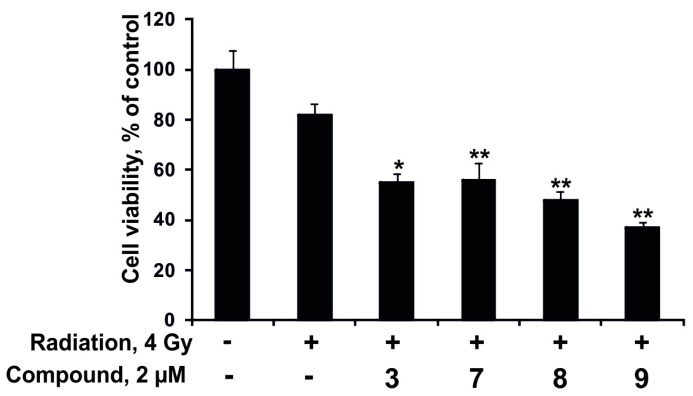
The effect of radioactive irradiation and a combination of radioactive irradiation and the compounds **3**, **7**–**9** on DLD-1 cancer cell proliferation. DLD-1 cells (8.0 × 10^3^) were treated with radiation 4 Gy and the compounds **3**, **7**–**9** (2 µM) for 96 h. Cell viability was estimated using the MTS assay. Data are represented as the mean ± SD as determined from triplicate experiments. A Student’s *t*-test was used to evaluate the data with the following significance levels: * *p* < 0.05, ** *p* < 0.01.

Nevertheless, the investigated compounds (2 µM) enhanced the antiproliferative effect from radioactive irradiation (4 Gy). Magnumoside C_4_ (**9**) possessed the highest activity in this experiment; it increased the inhibitory effect from radiation on proliferation of DLD-1 cancer cells by 45%. Magnumoside A_3_ (**3**), magnumoside C_1_ (**7**) and magnumoside C_2_ (**8**) enhanced the effect from radiation by more than 30% (Figure 7). Recently, it was reported that ginsenoside Rg3 isolated from the roots of *Panax ginseng* sensitized human lung carcinoma A549 and H1299 cells to γ-radiation and significantly enhanced the efficacy of radiation therapy in C57BL/6 mice bearing a Lewis lung carcinoma cell xenograft tumor [38]. Nevertheless, our finding of a synergism of the antiproliferative effect of the radioactive irradiation and a series of glycosides from *Neothyonidium magnum* on human tumor cells is the first study on sea cucumber triterpene glycosides.

## 3. Experimental Section

### 3.1. General Experimental Procedures

Specific rotations were measured on Perkin-Elmer 343 polarimeter. NMR spectra were obtained on an AVANCE III-700 Bruker spectrometer (Bruker BioSpin, Fällanden, Switzerland) at 700.13 (^1^H) and 176.04 (^13^C) MHz, δ in ppm rel. to Me_4_Si, *J* in Hz. ESI-MS/MS and HR-ESI-MS were run on an Agilent 6510 Q-TOF apparatus (Agilent Technologies, Santa Clara, CA, USA), sample concentration 0.01 mg/mL, in *m*/*z.* HPLC was carried out on an Agilent 1100 chromatograph equipped with a differential refractometer using Supelcosil LC-Si (4.6 × 150 mm, 5 μm) (Agilent Technologies, Santa Clara, CA, USA), Supelco Ascentis RP-Amide (10 × 250 mm, 5 μm) (Supelco Analytical, Bellefonte, PA, USA) and Diasfer *C-8* (4 × 250 mm, 5 μm) (Supelco Analytical, Bellefonte, PA, USA) columns. Polychrom-1 (powdered Teflon, 0.25–0.50 mm; Biolar, Olaine, Latvia), silica gel KSK (50–160 μM, Sorbpolimer, Krasnodar, Russia) were used for column chromatography.

### 3.2. Animal Material

Specimens of the sea cucumber *Neothyonidium* (=*Massinium*) *magnum* (family Phyllophoridae; order Dendrochirotida) were collected during the expedition of Joint Russia-Vietnam laboratory of Marine Biology RAS-VAST in South China Sea in Nha Trang bay near Tam Island. Sampling was performed by SCUBA in July 2015 (collector T. Dautova) at a depth of 8 m. Sea cucumbers were identified by T. Dautova; voucher specimens are preserved in the collection of the Museum of A.V. Zhirmunsky Institute of Marine Biology, Vladivostok, Russia.

### 3.3. Extraction and Isolation

The sea cucumbers were minced and extracted twice with refluxing 60% EtOH. The dry weight of the residue was about 59.2 g. The combined extracts were concentrated to dryness in vacuum, dissolved in H_2_O, and chromatographed on a *Polychrom-1* column (powdered Teflon, Biolar, Latvia). Eluting first of the inorganic salts and impurities with H_2_O and then the glycosides with 50% EtOH gave 1800 mg of crude glycoside fraction. Then it was chromatographed on Si gel column using CHCl_3_/EtOH/H_2_O (100:100:17) followed by (100:125:25) as mobile phase to give fractions 1 (332 mg) and 2 (340 mg). The fraction 1 was additionally chromatographed on Si gel column with CHCl_3_/EtOH/H_2_O (100:75:10) as mobile phase to give three subfractions: 1.1 (95 mg), 1.2 (127 mg) and 1.3 (46 mg). Subfraction 1.1 was submitted to HPLC on silica-based column Supelcosil LC-Si (4.6 × 150 mm) with CHCl_3_/MeOH/H_2_O (65:20:2) as mobile phase to obtain fractions 1.1.1 (45 mg) and 1.1.2 (5 mg) that were sequentially submitted to HPLC on a reversed-phase semipreparative Supelco Ascentis RP-Amide column (10 × 250 mm) with different ratios of MeOH/H_2_O/NH_4_OAc (1 M water solution) as mobile phase. Fraction 1.1.1 chromatographed with the ratio (65/34/1) followed by the ratios (63/36/1) gave magnumosides A_3_ (3) (3.7 mg) and A_4_ (4) (8.0 mg); (58/41/1)—magnumoside A_1_ (1) (3.6 mg). Fraction 1.1.2 chromatographed with the ratio (55/44/1) gave magnumoside A_2_ (2) (5.0 mg).

Subfraction 1.3, obtained after chromatography on Si gel, was submitted to HPLC on Supelco Ascentis RP-Amide column (10 × 250 mm) with MeOH/H_2_O/NH_4_OAc (1 M water solution) (57/41/2) followed by (55/43/2) as mobile phase to give magnumosides B_1_ (**5**) (2.6 mg), B_2_ (**6**) (1.8 mg) and colochiroside B_2_ (**10**) (2.0 mg).

The most polar fraction 2, obtained after the separation of crude glycoside fraction of Si gel column, was submitted to HPLC on Supelco Ascentis RP-Amide column (10 × 250 mm) with MeOH/H_2_O/NH_4_OAc (1 M water solution) (57/41/2) to give a set of subfractions. Subfraction 2.1 was submitted to HPLC on Diasfer C-8 (4.6 × 250 mm) column with AcN/H_2_O/ NH_4_OAc (1 M water solution) (24/75/1) to obtain magnumoside C_1_ (**7**) (5.7 mg). Subfraction 2.2 was submitted to HPLC in the same conditions to obtain magnumoside C_2_ (**8**) (2.5 mg) and C_4_ (**9**) (15 mg).

#### 3.3.1. Magnumoside A_1_ (**1**)

Colorless powder; $[\alpha {]}_{D}^{20}$ −48 (*c* 0.1, 50% MeOH); ^1^H NMR data see Table 1 and Table 2; ^13^C NMR data see Table 1 and Table 3; (−)HR-ESI-MS, *m*/*z*: 843.3838 [M_Na_ − Na]^−^ (calcd for C_41_H_63_O_16_S^−^, 843.3842); (−)ESI-MS/MS, *m*/*z*: 843.4 [M_Na_ − Na]^−^, 741.3 [M_Na_ − Na − SO_3_Na + H]^−^, 697.3 [M_Na_ − Na − Qui + H]^−^, 641.3 [M_Na_ − Na − C_10_H_18_O_4_]^−^, 497.2 [M_Na_ − Na − C_21_H_30_O_4_]^−^. (+)ESI-MS/MS, *m*/*z*: 889.4 [M_Na_ + Na]^+^, 769.4 [M_Na_ + Na − NaHSO_4_]^+^, 623.4 [M_Na_ + Na − NaSO_4_ − Qui]^+^, 491.3 [M_Na_ + Na − NaSO_4_ − Qui − Xyl]^+^.

#### 3.3.2. Magnumoside A_2_ (**2**)

Colorless powder; $[\alpha {]}_{D}^{20}$ −12 (*c* 0.1, 50% MeOH); ^1^H NMR data see Table 1 and Table 2; ^13^C NMR data see Table 1 and Table 3; (−)HR-ESI-MS, *m*/*z*: 843.3838 [M_Na_ − Na]^−^**,** (calcd for C_41_H_63_O_16_S^−^, 843.3842), (+)ESI-MS/MS, *m*/*z*: 889.4 [M_Na_ + Na]^+^, 769.4 [M_Na_ + Na − NaHSO_4_]^+^, 623.4 [M_Na_ + Na − NaSO_4_ − Qui]^+^, 491.3 [M_Na_ + Na − NaSO_4_ − Qui − Xyl]^+^; (−)ESI-MS/MS, *m*/*z*: 843.4 [M_Na_ − Na]^−^**,** 697.3 [M_Na_ − Na − Qui + H]^−^**,** 743.3 [M_Na_ − Na − C_6_H_12_O]^−^, 641.3 [M_Na_ − Na − C_10_H_18_O_4_]^−^, 497.2 [M_Na_ − Na − C_21_H_30_O_4_]^−^.

#### 3.3.3. Magnumoside A_3_ (**3**)

Colorless powder; $[\alpha {]}_{D}^{20}$ −63 (*c* 0.1, 50% MeOH); ^1^H NMR data see Table 1 and Table 2; ^13^C NMR data see Table 1 and Table 3. (−)HR-ESI-MS, *m*/*z*: 827.3892 [M_Na_ − Na]^−^ (calcd for C_41_H_63_O_15_S^−^, 827.3893); (+)ESI-MS/MS, *m*/*z*: 873.4 [M_Na_ + Na]^+^, 771.5 [M_Na_ + Na − NaSO_3_ + H]^+^, 753.5 [M_Na_ + Na − NaHSO_4_]^+^, 727.3 [M_Na_ + Na − Qui + H]^+^, 607.4 [M_Na_ + Na − NaSO_4_ − Qui]^+^, 475.3 [M_Na_ + Na − NaSO_4_ − Qui − Xyl]^+^; (−)ESI-MS/MS, *m*/*z* 641.3 [M_Na_ − Na − C_10_H_18_O_3_]^−^, 497.2 [M_Na_ − Na − C_21_H_30_O_3_]^−^.

#### 3.3.4. Magnumoside A_4_ (**4**)

Colorless powder; $[\alpha {]}_{D}^{20}$ −98 (*c* 0.1, 50% MeOH); ^1^H NMR: Table 1 and Table 2; ^13^C NMR data see Table 1 and Table 3; (−)HR-ESI-MS, *m*/*z*: 827.3892 [M_Na_ − Na]^−^ (calcd for C_41_H_63_O_15_S^−^, 827.3893); (+)ESI-MS/MS, *m*/*z*: 873.4 [M_Na_ + Na]^+^, 771.5 [M_Na_ + Na − NaSO_3_ + H]^+^, 753.5 [M_Na_ + Na − NaHSO_4_]^+^, 727.3 [M_Na_ + Na − Qui + H]^+^, 607.4 [M_Na_ + Na − NaSO_4_ − Qui]^+^, 475.3 [M_Na_ + Na − NaSO_4_ − Qui − Xyl]^+^; (−)ESI-MS/MS, *m*/*z* 641.3 [M_Na_ − Na − C_10_H_18_O_3_]^−^, 497.2 [M_Na_ − Na − C_21_H_30_O_3_]^−^.

#### 3.3.5. Magnumoside B_1_ (**5**)

Colorless powder; $[\alpha {]}_{D}^{20}$ −74 (*c* 0.1, 50% MeOH); ^1^H NMR data see Table 1 and Table 2; ^13^C NMR data see Table 1 and Table 3; (−)HR-ESI-MS, *m*/*z*: 1151.4954 [M_Na_ − Na]^−^ (calcd for C_53_H_83_O_25_S^−^, 1151.4950); (−)ESI-MS/MS, *m*/*z*: 1151.4 [M_Na_ − Na]^−^, 975.4 [M_Na_ − Na − MeGlc + H]^−^, 843.4 [M_Na_ − Na − MeGlc − Xyl]^−^, 697.3 [M_Na_ − Na − MeGlc − Xyl − Qui + H]^−^, 805.3 [M_Na_ − Na − C_21_H_30_O_4_]^−^, 629.2 [M_Na_ − Na − C_21_H_30_O_4_ − MeGlc + H]^−^, 497.2 [M_Na_ − Na − C_21_H_30_O_4_ − MeGlc − Xyl]^−^, 351.1 [M_Na_ − Na − C_21_H_30_O_4_ − MeGlc − Xyl − Qui + H]^−^.

#### 3.3.6. Magnumoside B_2_ (**6**)

Colorless powder; $[\alpha {]}_{D}^{20}$ −57 (*c* 0.1, 50% MeOH); ^1^H NMR: Table 1 and Table 2; ^13^C NMR data see Table 1 and Table 3; (−)HR-ESI-MS, *m*/*z*: 1151.4954 [M_Na_ − Na]^−^ (calcd for C_53_H_83_O_25_S^−^, 1151.4950); (−)ESI-MS/MS, *m*/*z*: 1151.4 [M_Na_ − Na]^−^, 975.4 [M_Na_ − Na − MeGlc + H]^−^, 843.4 [M_Na_ − Na − MeGlc − Xyl]^−^, 697.3 [M_Na_ − Na − MeGlc − Xyl − Qui + H]^−^, 805.3 [M_Na_ − Na − C_21_H_30_O_4_]^−^, 665.2 [M_Na_ − Na − C_30_H_45_O_5_]^−^.

#### 3.3.7. Magnumoside C_1_ (**7**)

Colorless powder; $[\alpha {]}_{D}^{20}$ −22 (*c* 0.1, 50% MeOH); ^1^H NMR: Table 1 and Table 2 (for carbohydrate chain); ^13^C NMR data see Table 1 and Table 3; (−)HR-ESI-MS, *m*/*z*: 1253.4328 [M_2Na_ − Na]^−^ (calcd for C_53_H_82_O_28_S_2_Na^−^, 1253.4337); (−)ESI-MS/MS, *m*/*z*: 1133.4 [M_2Na_ − Na − NaHSO_4_]^−^**,** 957.4 [M_2Na_ − Na − NaHSO_4_ − MeGlc]^−^,825.4 [M_2Na_ − Na − NaHSO_4_ − MeGlc − Xyl]^−^, 679.3 [M_2Na_ − Na − NaHSO_4_ −MeGlc − Xyl − Qui]^−^, 805.3 [M_2Na_ − Na − NaSO_3_ − C_21_H_30_O_4_ + H]^−^, 665.2 [M_2Na_ − Na − NaSO_3_ − C_30_H_45_O_5_]^−^, 533.1 [M_2Na_ − Na − NaSO_3_ − C_30_H_45_O_5_ − Xyl]^−^, 387.1 [M_2Na_ − Na − NaSO_3_ − C_30_H_45_O_5_ − Xyl − Qui]^−^, 255 [M_2Na_ − Na − NaSO_3_ − C_30_H_45_O_5_ − Xyl − Qui − Xyl]^−^.

#### 3.3.8. Magnumoside C_2_ (**8**)

Colorless powder; $[\alpha {]}_{D}^{20}$ −64 (*c* 0.1, 50% MeOH); ^1^H NMR data see Table 1 and Table 2; ^13^C NMR data see Table 1 and Table 3; (−)HR-ESI-MS, *m*/*z*: 1253.4328 [M_2Na_ − Na]^−^ (calcd for C_53_H_82_O_28_S_2_Na^−^, 1253.4337); (−)ESI-MS/MS, *m*/*z*: 1133.4 [M_2Na_ − Na − NaHSO_4_]^−^**,** 957.4 [M_2Na_ − Na − NaHSO_4_ − MeGlc]^−^,825.4 [M_2Na_ − Na − NaHSO_4_ − MeGlc − Xyl]^−^, 679.3 [M_2Na_ − Na − NaHSO_4_ −MeGlc − Xyl − Qui]^−^, 805.3 [M_2Na_ − Na − NaSO_3_ − C_21_H_30_O_4_ + H]^−^, 665.2 [M_2Na_ − Na − NaSO_3_ − C_30_H_45_O_5_]^−^, 533.1 [M_2Na_ − Na − NaSO_3_ − C_30_H_45_O_5_ − Xyl]^−^, 387.1 [M_2Na_ − Na − NaSO_3_ − C_30_H_45_O_5_ − Xyl − Qui]^−^, 255 [M_2Na_ − Na − NaSO_3_ − C_30_H_45_O_5_ − Xyl − Qui − Xyl]^−^.

#### 3.3.9. Magnumoside C_4_ (**9**)

Colorless powder. $[\alpha {]}_{D}^{20}$ −52 (*c* 0.1, H_2_O). NMR: See Table 2 and Table 3. HR ESI MS (−) *m*/*z*: 1237.4390 (calc. 1237.4388) [M_2Na_ − Na]^−^; ESI MS/MS (−) *m*/*z*: 1135.4 [M_2Na_ − Na − NaSO_3_ + H]^−^, 1117.4 [M_2Na_ − Na − NaHSO_4_]^−^, 959.4 [M_2Na_ − Na − NaSO_4_ − MeGlc]^−^, 827.4 [M_2Na_ − Na − NaSO_4_ − MeGlc − Xyl]^−^, 681.3 [M_2Na_ − Na − NaSO_4_ −MeGlc − Xyl − Qui]^−^, 665.2 [M_2Na_ − Na − NaSO_3_ − C_30_H_45_O_4_]^−^, 533.1 [M_2Na_ − Na − NaSO_3_ − C_30_H_45_O_4_ − Xyl]^−^, 387.1 [M_2Na_ − Na − NaSO_3_ − C_30_H_45_O_4_ − Xyl − Qui]^−^, 255 [M_2Na_ − Na − NaSO_3_ − C_30_H_45_O_4_ − Xyl − Qui − Xyl]^−^.

### 3.4. Preparation of the MTPA Esters of Compound ***2***

Two aliquots (0.6 mg) of compound **2** were treated with (−)-(*R*)- and (+)-(*S*)-α-methoxy-α-(trifluoromethyl)-phenylacetyl (MTPA) chloride (10 μL) in dry C_5_D_5_N (600 μL) for 2 h at r.t. in NMR tubes to give corresponding (*S*)- and (*R*)-MTPA esters. Data of 24-(*S*)-MTPA Ester of **2**. ^1^H NMR (700 MHz): 1.66 (m, 1H of CH_2_(22)), 1.79 (s, Me(27)), 1.85 (m, 1H of CH_2_(22)), 2.07 (m, 1H of CH_2_(23)), 2.23 (m, 1H of CH_2_(23)), 5.04 (brs, 1H of CH_2_(26)), 5.24 (brs, 1H of CH_2_(26)), 5.75 (m, H-C(24)). Data of 24-(R)-MTPA Ester of **2**. ^1^H NMR (700 MHz): 1.67 (s, Me(27)), 1.80 (m, 1H of CH_2_(22)), 1.98 (m, 1H of CH_2_(22)), 2.13 (m, 1H of CH_2_(23)), 2.27 (m, 1H of CH_2_(23)), 4.99 (brs, 1H of CH_2_(26)), 5.14 (brs, 1H of CH_2_(26)), 5.72 (m, H-C(24)).

### 3.5. Bioassay

#### 3.5.1. Cell Culture

The spleenocytes from CD-1 line mice were used. The spleen was isolated from mice and homogenized. The spleenocytes were washed thrice and resuspended with RPMI-1640 medium contained gentamicine 8 μg/mL (Biolot, Saint Petersburg, Russia). The museum tetraploid strain of murine ascites Ehrlich carcinoma cells from the All-Russian cancer center RAMS (Moscow, Russia) was used. The cells of the ascites Ehrlich carcinoma were separated from ascites, which were collected on day 7 after inoculation in mouse CD-1 line. The cells were washed of ascites thrice and then resuspended in liquid media DMEM with L-Glutamine (Biolot, Saint Petersburg, Russia).

#### 3.5.2. Cytotoxic Activity

The solutions of tested substances in different concentrations (20 µL) and cell suspension (200 µL) were added in wells of 96-well plate and incubated over night at 37 °C and 5% CO_2_. After incubation the cells were sedimented by centrifugation, 200 µL of medium from each well were collected and 100 µL of pure medium were added. Then 10 µL of MTT solution 5 µg/mL (Sigma, St. Louis, MO, USA) were added in each well. Plate was incubated 4 h, after that 100 µL SDS-HCl were added to each well and plate was incubated at 37 °C 4–18 h. Optical density was measured at 570 nm and 630–690 nm. The activity of the substances was calculated as the ratio of the dead cells to general cells amount (ED_50_). Typicoside A_1_ [28] and cucumarioside A_2_-2 [11] were used as positive controls.

#### 3.5.3. Hemolytic Activity

Blood was taken from a CD-1 mouse. The erythrocytes were washed thrice with 0.9% NaCl, centrifuged (450 g) on a centrifuge LABOFUGE 400R (Heraeus, Hanau, Germany) for 5 min followed by re-suspending in phosphate-buffered saline (PBS), pH 7.2–7.4. Erythrocytes were used at a concentration provided an optical density of 1.5 at 700 nm for a non-hemolyzed sample. 20 μL of a water solution of test substance with a fixed concentration (0.12–100.00 μM) were added to a well of a 96-well plate containing 180 mL of the erythrocyte suspension and incubated for 1 h at 37 °C. The plates were centrifuged (900× *g*) on a LMC-3000 laboratory centrifuge (Biosan, Riga, Latvia) for 10 min. 10 μL of the supernatant were placed to special microplate with plate bottom for determination of the optical density on a spectrofotometer *Multiskan FC* at λ = 570 nm. ED_50_ was calculated using SigmaPlot 3.02 software (Jandel Scientific, San Rafael, CA, USA). Triton X-100 (Biolot, Saint Petersburg, Russia) at concentration 1%, caused the hemolysis of 100% cells was used as positive control. The erythrocyte suspension in phosphate-buffered saline, pH 7.2–7.4 (PBS) with 20 μL of the solvent without a tested compound was used as negative control.

#### 3.5.4. DLD-1 Human Colorectal Adenocarcinoma Cell Culture

Human colorectal adenocarcinoma cells DLD-1 were cultured in RPMI-1640 medium. Culture media was supplemented with 10% fetal bovine serum (FBS) and penicillin—streptomycin solution. Cells were maintained in a sterile environment and kept in an incubator at 5% CO_2_ and 37 °C to promote growth. DLD-1 cells were sub-cultured every 3–4 days by their rinsing with phosphate buffered saline (PBS), adding trypsin to detach the cells from the tissue culture flask, and transferring 10–20% of the harvested cells to a new flask containing fresh growth media.

#### 3.5.5. Cytotoxicity against DLD-1 Cells Assay

DLD-1 cells (1.0 × 10^4^/well) were seeded in 96-well plates for 24 h at 37 °C in 5% CO_2_ incubator. The cells were treated with the compounds **1**–**9** at concentrations range from 0 to 100 µM for additional 24 h. Subsequently, cells were incubated with 15 µL MTS reagent for 3 h, and the absorbance of each well was measured at 490/630 nm using microplate reader “Power Wave XS” (Bio Tek, Winooski, VT, USA). All the experiments were repeated three times, and the mean absorbance values were calculated. The results are expressed as the percentage of inhibition that produced a reduction in absorbance by compound’s treatment compared to the non-treated cells (control).

#### 3.5.6. Soft Agar Assay

DLD-1 cells (8.0 × 10^3^) were seeded in 6-well plate and treated with the **3, 7**–**9** (10 µM) in 1 mL of 0.3% Basal Medium Eagle (BME) agar containing 10% FBS, 2 mM l-glutamine, and 25 µg/mL gentamicin. The cultures were maintained at 37 °C in a 5% CO_2_ incubator for 14 days, and the cell’s colonies were scored using a microscope “Motic AE 20” (Scientific Instrument Company, Campbell, CA, USA) and the Motic Image Plus computer program (Scientific Instrument Company, Campbell, CA, USA) [39].

#### 3.5.7. DLD-1 Cell Proliferation Assay

DLD-1 cells (8 × 10^3^) cells were seeded in 96-well plates in 200 mL of RPMI-1640/10%FBS medium at 37 °C in a 5% CO_2_ incubator for 24 h. Then the cells were treated with 2 µM of **3**, **7**–**9** for additional 24, 48, 72 or 96 h at 37 °C in a 5% CO_2_ atmosphere. The reaction was terminated by adding MTS reagent to each well as described in the Section 3.5.5.

#### 3.5.8. Radiation Exposure

Irradiation was delivered at room temperature using single doses of X-ray system XPERT 80 (KUB Technologies, Inc, Milford, CT, USA). The doses were from 4 to 8 Gy for colony formation assay. The absorber dose was measured using X-ray radiation clinical dosimeter DRK-1 (Akselbant, Moscow, Russia).

#### 3.5.9. Cell Irradiation

DLD-1 cells (5.0 × 10^5^) were plated at 60 mm dishes and incubated for 24 h. After the incubation, the cells were cultured in the presence or absence of 2 μM **3**, **7**–**9** for additional 24 h before irradiation at the dose of 4 Gy. Immediately after irradiation, cells were returned to the incubator for recovery. Three hour later, the cells were harvested and used for soft agar assay or proliferation assay to establish the synergism of radioactive irradiation and investigated compounds effects on colony formation or proliferation of tested cells.

#### 3.5.10. Statistical Analysis

All assays were performed in triplicate. The results are expressed as the means ± standard deviation (SD). A Student’s *t*-test was used to evaluate the data with the significance level of *p* < 0.05. The mean and standard deviation were calculated and plotted using SigmaPlot 3.02 Software (Jandel Scientific, San Rafael, CA, USA).

## 4. Conclusions

Summarizing the obtained data, three types of previously known carbohydrate chains, five new aglycones as well as one known aglycone have been found in the isolated glycosides 1–9. Magnumoside A_1_ (1) has uncommon non-holostane aglycone with a unique for sea cucumber triterpene glycosides 20(24)-epoxy-25-hydroxy-fragment in the side chain. The biosythesis of the glycosides found in *N. magnum* has mosaic character, i.e., transformation in cyclic systems of aglycones and in their side chains as well as in carbohydrate chains (elongation and sulfation) proceed parallel and independently and they have the characteristics of a biosynthetic network. Disulfated glycosides 7 and 8 showed surprisingly high hemolytic and cytotoxic actions in spite of the presence of hydroxyl-groups in their side chains. The data concerning cytotoxic activities on DLD-1 human colorectal adenocarcinoma cells good correlated with the data on mouse ascites Echrlich carcinoma cells that confirmed the usefulness of the last model tumor for screening of substances that are cytotoxic against human tumor cells. The data concerning synergy of the activities of the glycosides 3 and 7–9 in subcytotoxic doses and subtoxic doses of radiation were obtained for the first time where magnumoside C_4_ (9) revealed the highest increase of the inhibitory effect of radiation on cell proliferation of 45%. The substances having such effects allow a decrease in the effective doses of radiation that may be used for radiation therapy of human tumors. The search for substances with a similar mode of action among sea cucumber triterpene glycosides should be continued.

## Figures and Tables

**Table 1:** ^13^C and ^1^H NMR chemical shifts of carbohydrate moieties of magnumosides of the group A (**1**–**4**), B (**5**, **6**) and C (**7**–**9**) in C_5_D_5_N/D_2_O (5:1), δ in ppm, *J* in Hz.

Position	1–4, δC ^a^	1–4, δH ^b^	5, 6, δC ^a^	5, 6, δH ^b^	7–9, δC ^a^	7–9, δH ^b^
Xyl (1→C-3)						
1	104.8 CH	4.66 (d, 7.0)	104.8 CH	4.65 (d, 7.3)	104.8 CH	4.65 (d, 7.0)
2	**82.3 CH**	4.00 (t, 8.7)	**82.2 CH**	4.00 (t, 8.7)	**82.3 CH**	3.98 (t, 8.8)
3	75.1 CH	4.24 (t, 8.7)	75.2 CH	4.25 (t, 8.7)	75.0 CH	4.24 (t, 8.8)
4	*76.1 CH*	5.01 m	*76.0 CH*	5.04 m	*76.1 CH*	4.99 m
5	63.9 CH_2_	4.77 (dd, 5.2; 11.7)	63.9 CH_2_	4.77 (dd, 5.5; 11.9)	63.9 CH_2_	4.76 (dd, 5.4; 11.5)
		3.72 (t, 11.7)		3.71 (dd, 9.6; 11.9)		3.71 (t, 11.8)
Qui (1→2Xyl)						
1	105.2 CH	5.00 (d, 7.6)	104.7 CH	5.00 (d, 7.8)	104.7 CH	4.98 (d, 7.7)
2	76.4 CH	3.90 (t, 9.3)	75.8 CH	3.88 (t, 9.1)	75.8 CH	3.87 (t, 8.6)
3	76.8 CH	4.04 (t, 9.3)	74.7 CH	3.97 (t, 9.1)	74.8 CH	3.95 (t, 9.0)
4	**76.1 CH**	3.61 (t, 8.7)	**85.6 CH**	3.51 (t, 9.1)	**85.6 CH**	3.50 (t, 9.0)
5	72.8 CH	3.66 (dd, 5.8; 8.7)	71.3 CH	3.65 (dd, 5.9; 9.6)	71.4 CH	3.63 (dd, 6.1; 9.3)
6	18.1 CH_3_	1.53 (d, 5.9)	17.8 CH_3_	1.61 (d, 5.9)	17.8 CH_3_	1.60 (d, 6.1)
Xyl (1→4Qui)						
1			104.4 CH	4.77 (d, 7.7)	104.3 CH	4.76 (d, 7.7)
2			73.3 CH	3.89 (t, 8.6)	73.1 CH	3.85 (t, 8.6)
3			**86.4 CH**	4.12 (t, 8.6)	**87.0 CH**	4.04 (t, 8.8)
4			68.7 CH	3.94 m	68.7 CH	3.90 (t, 8.7)
5			65.8 CH	4.11 (dd, 5.6; 11.3)	65.7 CH	4.11 (dd, 5.3; 11.8)
				3.59 (t, 10.8)		3.59 (t, 11.3)
MeGlc (1→3Xyl)						
1			104.4 CH	5.21 (d, 8.0)	104.6 CH	5.12 (d, 7.9)
2			74.5 CH	3.88 (t, 8.7)	74.3 CH	3.80 (t, 9.4)
3			87.0 CH	3.68 (t, 8.6)	86.4 CH	3.64 (t, 9.4)
4			70.4 CH	3.89 m	69.9 CH	3.96 (t, 9.4)
5			77.5 CH	3.90 m	75.5 CH	4.03 m
6			61.7 CH_2_	4.38 (dd, 1.7; 11.5)	*67.1 CH_2_*	4.97 (d, 9.4)
				4.05 (dd, 5.9; 11.5)		4.72 (dd, 5.5; 11.0)
OMe			60.5 CH_3_	3.80 s	60.5 CH_3_	3.76 s

^a^ Multiplicity by DEPT; ^b^ Multiplicity by 1D TOCSY; Bold—interglycosidic bond; Italic—sulfation.

**Table 2:** ^1^H NMR data for the aglycones of compounds **1**–**9** in C_5_D_5_N/D_2_O, δ in ppm, *J* in Hz.

Position	1 ^a^	2, 6, 8 ^b^	3 ^b^	4, 9 ^a^	5, 7 ^b^
1	1.46 m	1.35 m	1.34 m	1.46 m	1.33 m
2	2.10 m, H*_α_*	1.94 m, H*_α_*	1.94 m, H*_α_*	2.09 m, H*_α_*	1.93 m, H*_α_*
	1.89 m, H*_β_*	1.74 m, H*_β_*	1.73 m, H*_β_*	1.87 m, H*_β_*	1.73 m, H*_β_*
3	3.26 (dd, 3.9, 11.6)	3.16 (dd, 3.8, 11.5)	3.16 (dd, 4.3, 11.7)	3.26 (dd, 4.1, 11.8)	3.16 (dd, 4.5, 11.4)
5	0.98 (dd, 3.3; 11.6)	0.86 (brd, 11.0)	0.86 (dd, 3.9; 11.7)	0.99 (dd, 3.6; 11.8)	0.86 (dd, 3.3; 11.9)
6	2.06 m, H*_α_*	1.92 m, H*_α_*	1.91 m, H*_α_*	2.05 m, H*_α_*	1.91 m, H*_α_*
	1.96 m, H*_β_*	1.82 m, H*_β_*	1.81 m, H*_β_*	1.97 m, H*_β_*	1.81 m, H*_β_*
7	5.61 (dt, 2.3; 7.4)	5.57 m	5.59 (brd, 7.4)	5.63 (dt, 2.4; 7.3)	5.56 (brd, 7.5)
9	3.18 (brd, 13.7)	3.03 (brd, 15.2)	3.02 (brd, 14.9)	3.23 (brd, 13.5)	3.02 (brd, 14.7)
11	2.03 m, H*_β_*	1.93 m	1.94 m	2.03 m, H*_β_*	1.95 m
	1.51 m, H*_α_*	1.40 m	1.41 m	1.54 m, H*_α_*	1.42 m
12	2.47 m, H*_β_*	2.50 m, H*_β_*	2.50 m	2.62 m, H*_β_*	2.47 m, H*_β_*
	2.03 m, H*_α_*	2.14 m, H*_α_*	2.09 m	2.14 m, H*_α_*	2.10 m, H*_α_*
15	2.12 (d, 13.2, H*_β_*)	2.08 (d, 13.5, H*_β_*)	2.10 (d, 13.5, H*_β_*)	2.15 (d, 13.3, H*_β_*)	2.08 (d, 13.8, H*_β_*)
	1.89 (dd, 2.6; 13.5, H*_α_*)	2.01 (dd, 2.0; 13.3, H*_α_*)	2.05 (brd, 11.5, H*_α_*)	1.94 (dd, 2.6; 13.4, H*_α_*)	2.00 (dd, 2.6; 13.4, H*_α_*)
16	4.84 br s	5.12 br s	5.08 br s	5.05 (d, 1.6)	5.14 br s
17	2.48 s	2.72 s	2.69 s	2.63 s	2.77 s
19	1.04 s	0.91 s	0.91 s	1.05 s	0.91 s
21	1.32 s	1.46 s	1.44 s	1.50 s	1.47 s
22	2.02 m	2.05 m	1.70 m	1.83 m	2.50 (br d, 6.9)
	1.73 m	1.83 m	---	1.80 m	---
23	2.08 m	2.06 m	1.72 m	2.40 m	5.92 (d, 15.8)
	1.87 m	1.97 m	1.62 m	2.31 m	---
24	3.97 (dd, 5.6; 9.3)	4.33 (t, 6.0)	2.00 (t, 7.4)	5.27 (t, 7.1)	6.13 (dt, 6.9; 8.0; 15.8)
26	1.31 s	5.16 br s	4.74 br s	1.70 s	1.48 s
	---	4.87 br s	4.73 br s	---	---
27	1.42 s	1.82 s	1.65 s	1.63 s	1.48 s
30	1.15 s	0.97 s	0.97 s	1.15 s	0.97 s
31	1.31 s	1.16 s	1.16 s	1.31 s	1.16 s
32	1.36 s	1.37 s	1.40 s	1.41 s	1.39 s

^a^ ratio C_5_D_5_N/D_2_O (4:1); ^b^ ratio C_5_D_5_N/D_2_O (5:1).

**Table 3:** ^13^C NMR data for aglycones of compounds **1**–**8** in C_5_D_5_N/D_2_O, δ in ppm, mult.

Position	1 ^a^	2, 6, 8 ^b^	3 ^b^	4, 9 ^a^	5, 7 ^b^
1	35.8 CH_2_	35.6 CH_2_	35.6 CH_2_	35.8 CH_2_	35.6 CH_2_
2	26.9 CH_2_	26.7 CH_2_	26.7 CH_2_	26.9 CH_2_	26.7 CH_2_
3	88.8 CH	88.9 CH	89.0 CH	88.8 CH	89.0 CH
4	39.4 C	39.1 C	39.2 C	39.4 C	39.2 C
5	47.7 CH	47.5 CH	47.6 CH	47.6 CH	47.5 CH
6	23.2 CH_2_	23.1 CH_2_	23.1 CH_2_	23.2 CH_2_	23.1 CH_2_
7	122.4 CH	122.3 CH	122.4 CH	122.4 CH	122.4 CH
8	147.6 C	147.6 C	147.6 C	147.8 C	147.6 C
9	46.1 CH	45.9 CH	46.0 CH	46.0 CH	45.9 CH
10	35.5 C	35.3 C	35.4 C	35.5 C	35.4 C
11	21.8 CH_2_	21.7 CH_2_	21.7 CH_2_	21.9 CH_2_	21.7 CH_2_
12	20.1 CH_2_	20.1 CH_2_	20.2 CH_2_	20.4 CH_2_	20.1 CH_2_
13	54.9 C	54.6 C	54.7 C	54.6 C	54.7 C
14	45.6 C	45.9 C	46.0 C	45.8 C	46.0 C
15	44.4 CH_2_	44.2 CH_2_	44.3 CH_2_	44.5 CH_2_	44.2 CH_2_
16	79.6 CH	80.1 CH	80.1 CH	79.5 CH	80.1 CH
17	61.6 CH	62.2 CH	62.3 CH	62.2 CH	61.4 CH
18	180.9 C	182.6 C	182.8 C	181.6 C	182.8 C
19	23.9 CH_3_	23.7 CH_3_	23.8 CH_3_	23.9 CH_3_	23.8 CH_3_
20	81.9 C	71.3 C	71.5 C	71.1 C	71.7 C
21	28.0 CH_3_	26.0 CH_3_	26.0 CH_3_	26.5 CH_3_	26.4 CH_3_
22	37.7 CH_2_	38.6 CH_2_	42.0 CH_2_	42.9 CH_2_	45.8 CH_2_
23	26.8 CH_2_	29.4 CH_2_	21.7 CH_2_	22.7 CH_2_	142.8 CH
24	86.7 CH	75.4 CH	38.1 CH_2_	125.1 CH	121.9 CH
25	70.1 C	148.4 C	145.9 C	131.0 C	69.9 C
26	26.3 CH_3_	110.6 CH_2_	110.3 CH_2_	25.5 CH_3_	29.9 CH_3_
27	26.7 CH_3_	17.7 CH_3_	22.2 CH_3_	17.4 CH_3_	29.9 CH_3_
30	17.1 CH_3_	17.0 CH_3_	17.1 CH_3_	17.1 CH_3_	17.0 CH_3_
31	28.5 CH_3_	28.4 CH_3_	28.5 CH_3_	28.5 CH_3_	28.5 CH_3_
32	34.3 CH_3_	34.3 CH_3_	34.3 CH_3_	34.3 CH_3_	34.5 CH_3_

^a^ ratio C_5_D_5_N/D_2_O (4:1); ^b^ ratio C_5_D_5_N/D_2_O (5:1).

**Table 4:** Hemolytic activity of the glycosides **1**–**9** against mouse erythrocytes and cytotoxic activity against mouse spleenocytes and the ascites form of mouse Ehrlich carcinoma cells.

Compound	Hemolytic Activity, ED_50_, μM/mL *	Cytotoxic Activity, IC_50_, μM/mL **	
		spleenocytes	Ehrlich carcinoma cells
**1**	90.18 ± 1.57	>100.00	>100.00
**2**	33.33 ± 0.48	94.18 ± 1.18	>100.00
**3**	12.53 ± 0.29	19.20 ± 0.03	18.95 ± 0.03
**4**	20.12 ± 0.14	37.64 ± 0.00	28.37 ± 0.42
**5**	49.57 ± 0.63	>100.00	>100.00
**6**	58.11 ± 0.69	>100.00	>100.00
**7**	6.97 ± 0.14	8.97 ± 0.04	18.65 ± 0.00
**8**	16.20 ± 0.49	17.31 ± 0.43	37.52 ± 0.00
**9**	6.52 ± 0.16	12.23 ± 0.33	35.06 ± 0.18
**Typicosid A_1_**	3.15 ± 0.12	9.92 ± 0.12	35.66 ± 0.11
**Cucumarioside A_2_-2**	0.82 ± 0.02	1.21 ± 0.02	1.39 ± 0.015

* ED_50_ is the effective dose of compound causing 50% of hemolysis of cells; ** IC_50_ is a concentration of substance caused 50% reduction in cell viability.

**Table 5:** The cytotoxic activity of the glycosides **1**–**9** against DLD-1 cells.

Glycoside	IC_50_, µM *	Glycoside	IC_50_, µM *
magnumoside A_1_ (**1**)	>100	magnumoside B_2_ (**6**)	>100
magnumoside A_2_ (**2**)	>100	magnumoside C_1_ (**7**)	32.9
magnumoside A_3_ (**3**)	30.3	magnumoside C_2_ (**8**)	37.1
magnumoside A_4_ (**4**)	>100	magnumoside C_4_ (**9**)	33.9
magnumoside B_1_ (**5**)	>100	–	–

* IC_50_ is a concentration of substance caused 50% reduction in cell viability.
